# Supplementary figures and images for: Predicting nitroimidazole antibiotic resistance mutations in Mycobacterium tuberculosis with protein engineering
Source: PLoS Pathog. 2020 Feb 7;16(2):e1008287. doi: 10.1371/journal.ppat.1008287 (PMC7032734; doi:10.1371/journal.ppat.1008287)

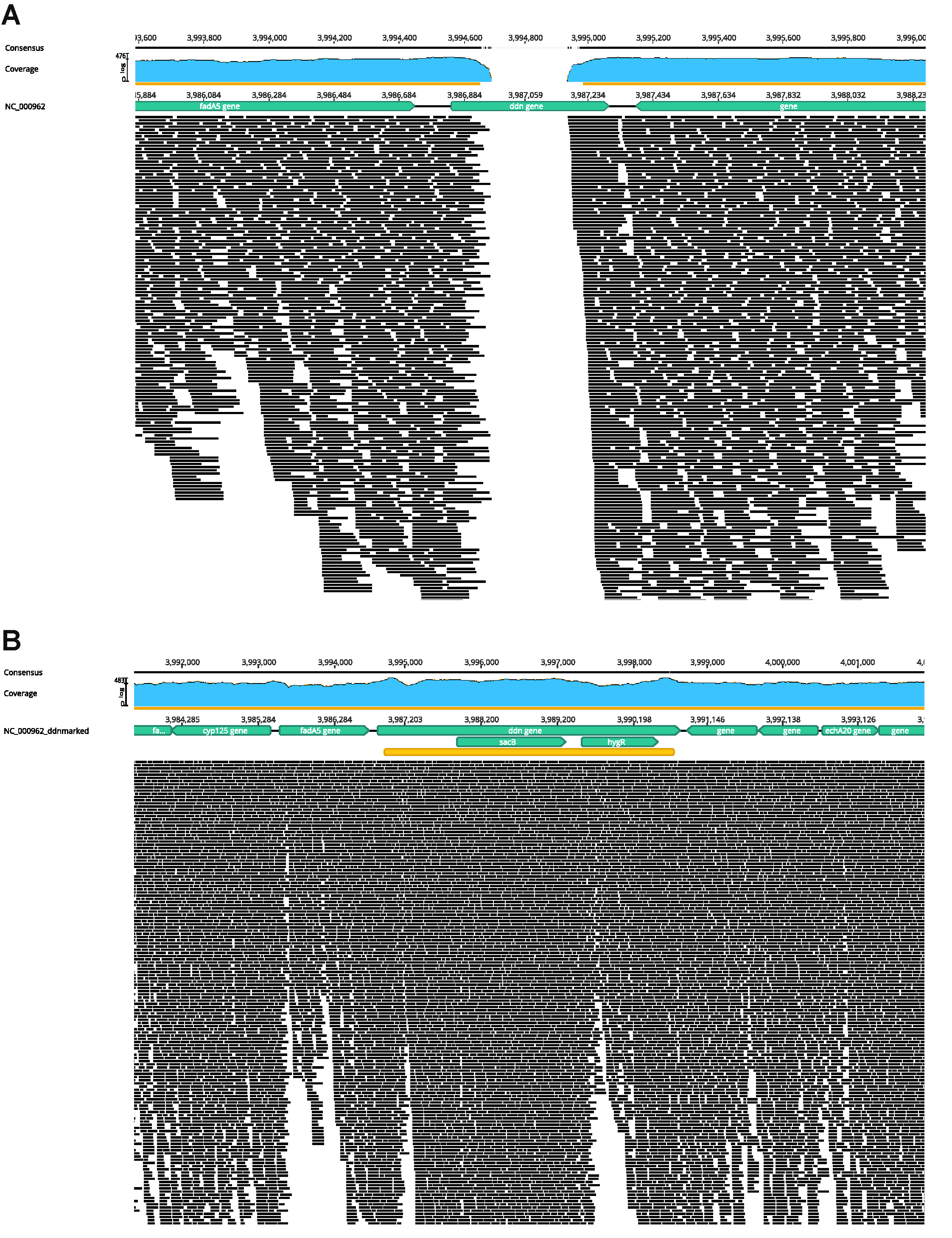

Supplement: S1 Fig — (A) Map of the Δddn mutant reads using Bowtie 2 to H37Rv genome. (B) Map of the Δddn mutant reads using Bowtie 2 to H37Rv genome with expected hygromycin marker. (TIF) [file ppat.1008287.s001.tif]
