# Supplementary material for: Predicting nitroimidazole antibiotic resistance mutations in Mycobacterium tuberculosis with protein engineering
Source: PLoS Pathog. 2020 Feb 7;16(2):e1008287. doi: 10.1371/journal.ppat.1008287 (PMC7032734; doi:10.1371/journal.ppat.1008287)
Supplement: Attachment — Submitted filename: Lee_Response_to _the _reviewers.docx [file ppat.1008287.s005.docx]

10 Dec 2019

Dear Professor Boshoff,

Please find attached our resubmitted article “**Predicting nitroimidazole antibiotic resistance mutations in *Mycobacterium tuberculosis* with protein engineering**” that we wish to be considered for publication in *Plos Pathogens*.

The comments from yourself and the reviewers were very helpful, and we think we have managed to improve the manuscript substantially.

There were two major aspects that we needed to improve:

**1: Defining the physiological role of Ddn:**

First, it is extremely difficult to define the physiological role of a conditionally non-essential enzyme like Ddn without any ambiguity. However, we have done our best in this situation and have three lines of evidence that it is likely involved in respiration, via its ability to reduce menaquinone to menaquinol. First, we have the *in vitro* enzyme kinetics. Although the activity is certainly at the low-end of the range, it is not outside the distribution of typical enzymes (Bar-Even *et al.* *Biochemistry* 2015, 54, 4969), while the *K*_M_ is significantly lower (better) than the majority of enzymes. This lower than expected turnover could be due to the unnatural conditions of the assay (they will natively associate with the membrane). To address this, we performed several additional experiments, to investigate the effects of Ddn on bioenergetics in mycobacterial membranes. Menaquinone is the primary electron carrier in these membranes, and we demonstrate that when Ddn and F_420_H_2_ are present the rate of O_2_ consumption is significantly increased – indicative of increased reduction of menaquinone. Finally, the fitness cost (described in more detail below) is seen when cells emerge from hypoxia – which is consistent with the previous results, given the increased oxidative stress and involvement of respiration (via menaquinone) in this process. This is clearly an important physiological process and we are only at the early stages of our understanding, but we hope this is sufficient for this study – which is primarily focused on predicting nitroimidazole antibiotic resistance mutations in *Mycobacterium tuberculosis* with protein engineering. In other words, we hope that you agree that we have established that Ddn most likely has an important physiological role that involves menaquinone.

**2: Establishing that loss of Ddn-activity has a fitness cost:**

To address this comprehensively, we have created a hygromycin marked deletion mutant in the mc^2^6230 strain of *Mycobacterium tuberculosis* using phage transduction. Using this strain, we tested the marked Δ*ddn* strain in competition with the wild type isogenic strain when reaerated from hypoxia after 37 and 70 days, measuring the relative proportion of wild-type and Δ*ddn* at 0, 7 and 17 day intervals. This showed conclusively that the absence of *ddn* lowers the fitness of *M. tuberculosis* when emerging from hypoxia. Given the importance of hypoxic survival to *M. tuberculosis*, we hope that this provides support for our proposal that mutants without functional Ddn will be less fit.

There were also a number of minor changes that were suggested – all of these changes have been made.

We have now made and tested (individually – not in a high throughput screen) 75 unique mutations in the 141 amino acid protein (~1/3 of the protein sequence), analysed 15,000 genomes of TB isolates, performed *in vivo* (mouse) experiments, membrane bioenergetics experiments, generated a Δ*ddn* strain and performed competition growth analysis experiments to estimate the effect on fitness in aerobic, hypoxic and reaerated conditions. In the context of the implications of these findings on public health and the increasing use of nitroimidazole prodrugs, we believe it is vital that these results are published rapidly in a journal of prominence such as *Plos Pathogens,* to allow them to reach a wide audience.

Best regards,

Colin Jackson

**Specific responses:**

The question whether this enzyme is a specific menaquinone reductase is relevant. Thus, it is warranted to probe a few substrates as suggested. In addition, it is important to note that fitness was never demonstrated. Measurement of fitness by a competitive assay under the relevant in vitro conditions could substantially strengthen this work. This can be done in vitro for the hypoxia work with the appending conclusions then limited to exit from hypoxia by performing fitness assessment (competition assay of fit versus impaired fitness mutants) with analysis done by allele-specific PCR. An ideal experiment would of course be competitive experiments in mice with for example 1:5 and 5:1 ratios to see which strain gets out-competed since this would really substantiate the claims about in vivo fitness.

We thank the reviewers and editor for this suggestion. As menaquinone is the only quinone in *M. tuberculosis*, rather than test exogenous substrates, we have performed membrane bioenergetics experiments, to show that Ddn increases respiration (*via* reduction of menaquinone). We have now also completed competition experiments (Figure 8), which do indeed substantiate the claims around fitness. We were unable to complete them in mice – we hope you understand that these are major experiments and we will focus on doing them for a follow-up paper. We hope that the additional 85-day competition experiments are sufficient. Future studies will focus on the mouse related work, but we hope that the substantial effort that has gone into the protein and *in vitro* work in this study is enough to merit its publication.

Reviewer's Responses to Questions

**Part II – Major Issues: Key Experiments Required for Acceptance**
Please use this section to detail the key new experiments or modifications of existing experiments that should be absolutely required to validate study conclusions.

Generally, there should be no more than 3 such required experiments or major modifications for a "Major Revision" recommendation. If more than 3 experiments are necessary to validate the study conclusions, then you are encouraged to recommend "Reject".


Reviewer #1: None

*Reviewer #2: The authors need to improve their description of the enzymology results and its interpretations.*

*For example, on the first paragraph of results, the authors "Ddn catalyzed menaquinone reduction in vitro with moderate efficiency (kcat/KM = 8.6 x 102 M-1 s-1)...". 10^2 M-1s-1 is not moderate efficiency, it is low efficiency. One would expect a value at around 10^6 M-1s-1 for a metabolic enzyme. Also, " and physiologically relevant affinity (KM = 22.4 ± 3.8 μM) (Table 1);", what is the concentration of menaquinone in the cells? Only by knowning that, the authors can judge if this is or isn't physiologic. Finally, Km does not equal to Kd, in most cases I have seen. Therefore, the authors must swap "affinity" by "Km".*

We agree with this and have modified these sentences (ln 135-144).

“Ddn catalyzed menaquinone-1 reduction *in vitro* with a *k*_cat_/*K*_M_ value of ~10^3^ M^-1^ s^-1^ and a *K*_M_ of 22.4 ± 3.8 µM **(Figure 1a, Table 1).** It should be noted that the catalytic rate is likely to be higher in the crowded native environment of the *M. tuberculosis* cell where Ddn and menaquinone are co-localised at the cell membrane [25]. The enzyme kinetics of Ddn are comparable to another mycobacterial menaquinone oxidoreductase, MenJ (Rv0561c), which is found in the menaquinone biosynthetic pathway (*k*_cat_/*K*_M_ of ~10^4^ M^-1^ s^-1^ and *K*_M_ of 30 µM [24]). The *K*_M_ value is significantly lower than the median *K*_M_ of enzymes with their native substrates (~130 µM), and the *k*_cat_/*K*_M_ value is within the broad distribution of known enzymes (10^3^-10^7^ M^-1^ s^-1^), albeit at the lower end of the range ([26]).”

The additional references that we have included show that it is comparable to other characterized menaquinone oxidoreductases in terms of *k*_cat_/*K*_M_ and is at the low end of the normal distribution of *k*_cat_/*K*_M_ values for enzymes.

On the second paragraph of the results, the authors leap to a conclusion without any supporting data, by stating "Having established that Ddn is a menaquinone reductase,...". To this point, the authors demonstrated that Ddn can reduce menaquinone, but that does not prove that Ddn is a menaquinone reductase. To prove that, the authors must test a number of other substrates for enzymes that reduce quinone and show that kcat/Km values are smaller than what they get for menaquinone. Showing that an enzyme act (very poorly) on a substrate does not prove anything. The authors must use a variety of other quinone reductase substrates to probe the substrate specificity and therefore assign the likely bona fide substrate specificity of Ddn, if that is what they are claiming they are doing.

We agree with the reviewer. We have changed the wording to be more cautious and now state (Ln 149-150):

“Having established that Ddn can reduce menaquinone with efficiency that could be physiologically relevant,”

Instead of finding activities with other substrates (menaquinone is the only native quinone in *M. tuberculosis* (Hards, Rodriguez, Cairns, & Cook, 2019; Hatfull & Jacobs Jr, 2014; Holsclaw et al., 2008), which are less relevant given they are outside the physiological environment of the enzyme, we instead investigated the effects of Ddn complementation on respiration in mycobacterial membranes (in which menaquinone/menaquinol is the primary electron carrier). This is described on lines 151-169.

“we then investigated whether the menaquinone reductase activity of Ddn was coupled to the mycobacterial respiratory chain by comparing the rates of respiratory oxygen consumption of *M. smegmatis* in the presence and absence of a complementation vector expressing *ddn*. We observed that the addition of glucose 6-phosphate (G6P), F_420_, and F_420_ dependent glucose-6-phosphate dehydrogenase (FGD), which catalyzes the reduction of F_420_ to F_420_H_2_ for use by F_420_H_2_-dependent enzymes such as Ddn [26], resulted in a statistically significant increase in oxygen consumption by mycobacterial membranes when the membranes are activated by NADH **(Fig. 1b)**. This was dependent on the presence of cytochrome *bd* oxidase, which utilizes reduced menaquinone (i.e. menaquinol) as an electron source **(Fig. 1b)**. The rate of NADH oxidation was not affected by the addition of F_420_H_2_ in the absence of *ddn*, which implies endogenous NADH-dependent oxidases do not contribute to the increased oxygen consumption **(Fig. 1c)**. This supports the idea that mycobacteria can couple F_420_H_2_ oxidation to O_2_ reduction through the respiratory chain *via* cytochrome *bd* oxidase and demonstrates that bacteria can use F_420_H_2_ as a respiratory electron donor. We also observed that the extent of oxygen consumption was significantly higher in membranes purified from the strain that expressed *ddn*, relative to empty vector controls **(Fig. 1B)**, which suggest Ddn serves as a menaquinone reductase in the respiratory chain, with the remaining stimulation attributable to F_420_H_2_ oxidation by native Ddn homologs of *M. smegmatis*. Altogether, these results suggest that Ddn likely plays a role in bacterial respiration through reduction of menaquinone.”

We hope the reviewer agrees that the combination of the *in vitro* activity and the membrane bioenergetics experiments provide sufficient evidence for us to suggest that Ddn “likely plays a role in bacterial respiration through reduction of menaquinone”.

Reviewer #3: This is an exceptional biochemical analysis of amino acid sequence - structure - function relationships in Ddn with respect to its native reductase activity and the ability to activate nitroimidazoles. The study is well-executed from a biochemical perspective and has important implications for surveillance of the reported mutations in the clinical setting which would ultimately assist in better stewardship of nitroimidazoles as therapeutic agents for TB. That said, there are some shortfalls, detailed below

1. The authors use the term fitness several times but make no substantive measure of fitness in laboratory experiments. Most conclusions are derived from clinical and epidemiological information. Several aspects may be at play in the clinical settings and these remarks should be reconsidered. The claims of fitness costs or lack thereof are not support by the data presented in this manuscript.

We have now completed 85-day competition experiments to demonstrate that *ddn* deletion mutations have reduced fitness, relative to WT when emerging from hypoxia (Figure 8 and Ln 316-337). This now supports the claim of fitness costs.

“To better understand the competitive cost that loss of Ddn function causes to *M. tuberculosis* during recovery from hypoxia, we created a hygromycin marked deletion mutant in the mc^2^6230 strain of mycobacterium tuberculosis using phage transduction. The suspected Δ*ddn* mutants with hygromycin resistance displayed MIC values against delamanid of greater than 512 μM. The deletion mutants were then confirmed using whole genome sequencing (Supplementary figure 1). The Δ*ddn* mutant displayed no growth or survival defects compared to the isogenic wild type when grown in aerobic or hypoxic conditions (Figure 8b and c) consistent with the previous results on *ddn* point mutants (Figure 8a). We then tested the marked Δ*ddn* strain in competition with the wild type isogenic strain when reaerated from hypoxia. As a control we took the day 7 aerobic growth sample of each strain and grew them in competition. We found that the Δ*ddn* mutant had lost no competitiveness when taken from aerobic conditions with the proportional percentage of Δ*ddn* remaining at about ~50% (Figure 8d). We then took equal proportions of each strain from hypoxia at 37 days and 70 days, which we grew in competition (Figure 8c). The proportion of Δ*ddn* was then measured at days 0, 7 and 17. We found that with the Day 37 hypoxic sample the Δ*ddn* mutant had an initial drop in proportion to 28% at 7 days but by day 17 the proportion of the Δ*ddn* mutant had returned to 57% (Figure 8d). The day 70 hypoxic sample had an initial drop off Δ*ddn* proportion to 33% which was maintained through until day 17 (Figure 8d). These data demonstrate that the Δ*ddn* mutant cannot compete with wild type after exit from prolonged hypoxia and that Ddn contributes to fitness under these conditions, perhaps because they are more susceptible to oxidative stress or have some defect in anaerobic respiration.”

2. Similarly, hypoxia seems to used interchangeably with dormancy. Dormancy is a complex phenomenon associated with metabolic quiescence. It is unclear if hypoxia in a test tube faithfully reflects this state. Moreover, recovery from such conditions in the lab is not a measure of fitness or transmissibility. This is all speculative and should be revised.

We have changed this throughout the manuscript in accordance with the suggestion of the reviewer (which we agree with). (ln 33, 57, 105, 119, 343, and 347)

**Part III – Minor Issues: Editorial and Data Presentation Modifications**
Please use this section for editorial suggestions as well as relatively minor modifications of existing data that would enhance clarity.

Reviewer #1:

1. One of the interesting and important points of the manuscript is that resistance to these new bicyclic nitroimidazoles potentially occurs through genetic drift rather than by selection. The authors might consider bringing this point into the title and abstract.

We have added “and confirmed, to be resistant to pretomanid, revealing that resistance to this drug has arisen through genetic drift and not selective pressure in this instance.” to the author summary (ln 61-62).

2. In author summary, change "Strikingly, when analysed >15,000 M. tuberculosis..." to "Strikingly, when we analysed >15,000 M. tuberculosis..."

This change has been made (ln 57-58).

3. "In order to spread effectively and endanger health, these resistant strains would need to retain sufficient fitness to survive all stages of the lifecycle of M. tuberculosis, including recovery from dormancy." Formal proof of the importance of dormancy for fitness of M. tuberculosis in the host is non-existent. We know that such populations exist, but, whether this mode is essential for transmission and pathogenesis is unknown. The authors should modify this sentence.

We have altered this sentence (ln 103-105) to “We speculate that in order to for tuberculosis to spread effectively and endanger health, these resistant strains would need to retain sufficient fitness to survive all stages of the lifecycle of *M. tuberculosis,* including recovery from hypoxia.”. We hope this is ok – it is not so controversial to suggest that recovery from hypoxia is important.

4. Change "M. tuberculosis H37Rv and M. marinum, which were the only two strains that encoded Ddn orthologs with in vitro pretomanid activation activity," to "M. tuberculosis (H37Rv) and M. marinum, which were the only two species that encoded Ddn orthologs with in vitro pretomanid activation activity,"

This change has been made (ln 190-191).

5. "Every mutant tested was able to reduce menadione, indicating that there was selective pressure to maintain the physiological function of Ddn." Probably true, but, without the isoprenyl tail, menadione may be a more promiscuous substrate, it is overreaching to state that this observation indicates maintenance of function, would need to test MQ (but not necessary to do this). May be better to state that it is consistent with selective pressure for maintenance of the physiological function of Ddn...

This change has been made (ln 206-208).

6. "could readily occur and spread with sufficient selection pressure." to "could readily occur and spread with sufficient selective pressure."

This change has been made (ln 255-256).

7. "...will result in substantial loss of fitness given that the ability to recover from dormancy is such an important aspect of M. tuberculosis pathogenesis." While this might be the case, to my knowledge, there is no direct evidence, and the references provided do not provide such evidence. Maintenance of this function supports its role in fitness, but, that role might be distinct from that which has been modeled in the laboratory.

We have rephrased this to “could result in substantial loss of fitness given that the ability to recover from hypoxia is an important aspect of *M. tuberculosis* pathogenesis [3,23,36–38].” (ln 346-348). Now that we have shown reduced fitness in emergence from hypoxia and enhancement of menaquinone reduction in mycobacterial membranes, we hope the reviewer agrees this is a reasonable level of discussion.

8. "The activation of the prodrugs pretomanid and delamanid by Ddn is a promiscuous activity that is not coupled to its native activity." Statement needs modification, hydride transfer is part of the native activity of Ddn

We have rephrased this to “is a promiscuous activity that is not fully coupled to its ability to bind and reduce its native substrate; i.e. loss of pretomanid activation is possible without total loss of the native activity.” (ln 354-356).

9. "It has long been known that promiscuous activities are more susceptible to mutation than native functions..." Modify, activities don't mutate.

This change has been made (ln 356-357).

10. Figure 3 title, "sequence" and "sequences" is redundant, for figure 3 legend, should describe what color represents.

This change has been made (ln 636-640).

11. Figure 4, increase font size of SNPs

We have increased the font size to the largest size possible before the labels start to overlap.

12. Table 2 seems redundant with figures that show these data. However, I do favor inclusion -perhaps as supplemental. Also, the meaning of no activity (below limit of detection?) and no expression is unclear.

We are comfortable with the editor deciding whether they prefer the tables in the main text or supporting information. We agree with the reviewer and have changed all reference to no activity to “below level of detection”.

13. Table 3, rate of 0 should be expressed as less than the limit of detection. Need to define N/A, not sure why it says N/A for M. marinum when Table 2 shows activity.

We agree with the reviewer and have changed all reference to no activity to “below level of detection”. The activity data for *M. marinum* has been added to table 3

14. Table 4 seems redundant with figure 4.

We are comfortable with the editor deciding whether they prefer the tables in the main text or supporting information.

15. Same data is shown in multiple figures and tables, most journals require footnotes for repeated data.

We have added footnotes, so that it is clear the tables contain the data used to produce the figures.

16. For modeling, please show top docking scores.

This data is now included in the figure caption (Figure 7).

17. Figure 7, replicates and statistics?

Figure 7a (now 8a) has replicates and error bars, Figure 7b did not. This is now replaced by the new data with the *ddn* deletion mutant, which has replicates and statistics.

18. Need to provide additional information for readers to understand what is presented in Table S2

We have modified the title to make the information clearer (ln 946-950).

19. Check th

This comment seems to have been cut off prematurely.

Reviewer #2: There are a few typos in the manuscript. For example, on Figure 1A (M. ulcerans, not M. ulceran) and the title of the figure (binding, not bindng).

These changes have been made (ln 623 and now fig 1b).

Please, change "Wt" by the strain name used, as for this case, clearly H37Rv or analogous strain is not a wild-type.

Everything has now been labelled as either H37Rv or mc^2^ 6230. We also used WT to refer to wild-type Ddn (which is confusing) – so we have now explicitly defined this as Ddn where appropriate (Fig 4 and 6).
